# An efficient *Foxtail mosaic virus *vector system with reduced environmental risk

**DOI:** 10.1186/1472-6750-10-88

**Published:** 2010-12-16

**Authors:** Zun Liu, Christopher M Kearney

**Affiliations:** 1 Department of Biology, Baylor University, One Bear Place #7388, Waco, TX, 76798 USA; 2 Endocrine Unit, Department of Medicine, Massachusetts General Hospital and Harvard Medical School, Boston, Massachusetts 02114 USA

## Abstract

**Background:**

Plant viral vectors offer high-yield expression of pharmaceutical and commercially important proteins with a minimum of cost and preparation time. The use of *Agrobacterium tumefaciens *has been introduced to deliver the viral vector as a transgene to each plant cell via a simple, nonsterile infiltration technique called "agroinoculation". With agroinoculation, a full length, systemically moving virus is no longer necessary for excellent protein yield, since the viral transgene is transcribed and replicates in every infiltrated cell. Viral genes may therefore be deleted to decrease the potential for accidental spread and persistence of the viral vector in the environment.

**Results:**

In this study, both the coat protein (CP) and triple gene block (TGB) genetic segments were eliminated from *Foxtail mosaic virus *to create the "FECT" vector series, comprising a deletion of 29% of the genome. This viral vector is highly crippled and expresses little or no marker gene within the inoculated leaf. However, when co-agroinoculated with a silencing suppressor (p19 or HcPro), FECT expressed GFP at 40% total soluble protein in the tobacco host, *Nicotiana benthamiana*. The modified FoMV vector retained the full-length replicase ORF, the TGB1 subgenomic RNA leader sequence and either 0, 22 or 40 bases of TGB1 ORF (in vectors FECT0, FECT22 and FECT40, respectively). As well as *N. benthamiana*, infection of legumes was demonstrated. Despite many attempts, expression of GFP via syringe agroinoculation of various grass species was very low, reflecting the low *Agrobacterium*-mediated transformation rate of monocots.

**Conclusions:**

The FECT/40 vector expresses foreign genes at a very high level, and yet has a greatly reduced biohazard potential. It can form no virions and can effectively replicate only in a plant with suppressed silencing.

## Background

Plant expression systems have been developed as production platforms for many therapeutic proteins over the past two decades. Although many foreign proteins have been expressed in stably transgenic plants, plant viral vectors have emerged as the most efficient approach to achieving high-level expression of recombinant proteins in plants [1,2]. These self-replicating vectors produce maximum levels of foreign gene expression and require minimal set-up time. It is often possible to generate purified recombinant protein within three weeks of receiving a gene sequence [3,4].

However, the potential widespread use of recombinant viruses raises concerns about possible risks to the environment. Bio-safety issues must be considered to prevent the spread of the genetically engineered virus from experimental plants to susceptible wild plants [5-7]. Intact viral vectors have the potential to spread and infect non-target plants, but replication-defective or movement-defective viruses avoid these problems. These deleted viral vectors also address cross-contamination issues in the growth room and greenhouse. In the field, it might be possible to achieve high expression in transgenic plants carrying an inducible virus as a transgene [8,9]. In all of these cases, deleted virus vectors would be greatly preferred over full virus vectors for reduced transmission and persistence.

An obvious disadvantage to the deleted virus approach is that the vector cannot spread past the originally inoculated cells. However, this weakness can be successfully overcome by the agroinoculation technique, which uses *Agrobacterium tumefaciens *to deliver the virus sequence, carried in a binary vector, to the genome of the vast majority of plant cells in the infiltration zone of the leaf using whole, nonsterile plants [10]. For small scale use, a syringe is used to infiltrate leaves with *Agrobacterium*, while for large scale applications, vacuum infiltration is used to inoculate an entire greenhouse at once [10]. For both agroinoculation and transgenic use, systemic spread becomes an unnecessary property. Agroinoculation involves the local transformation of the infiltrated leaf with the viral cDNA as a part of the T-DNA of the Ti plasmid. A plant promoter (most commonly CaMV 35S) placed upstream of the viral cDNA induces the transcription of viral genome in the plant nucleus and viral RNA is transported to cytoplasm for viral replication.

Over the past few years, several deleted viral vectors delivered by agroinoculation have been created and some are used commercially. *Tobacco mosaic virus *(TMV) lacking the coat protein (CP) gene has been used to express a large number of foreign proteins commercially [4,11,12]. Removal of the CP gene from TMV can lead to unexpectedly large increases in foreign gene expression [13]. In the *Potato virus X *(PVX) replacement virus vector, both the triple gene block (TGB) and CP viral genes were removed, leaving only the replicase gene and terminal untranslated regions, and these deleted genes were replaced with GFP [14]. The expression level of GFP from this vector was 2.5-fold higher than that of full-length PVX vector with the GFP encoding sequence between the triple gene block and the CP genes. A defective RNA TMV vector has also been shown to express at high levels [15].

*Agrobacterium *infiltration-mediated transient expression can be greatly enhanced by suppression of gene silencing. An RNA silencing suppressor, such as p19 [16] from tomato bushy stunt virus or HcPro [17] from potato virus Y, is co-inoculated in a separate strain of *Agrobacterium *along with the *Agrobacterium *carrying the viral cDNA. Using this approach, highly efficient production of GFP from a TMV-based vector was achieved with up to a 100-fold increase of the overexpression level [18]. As well, potexvirus expression was greatly increased with suppressor co-inoculation [14].

The FECT vectors are derived from foxtail mosaic virus (FoMV) which is a member of the genus *Potexvirus. Potexvirus *is a large group of flexuous and filamentous plant viruses with a single-stranded, positive-sense genomic RNA which has a cap structure at the 5' terminus and a poly-(A) tail at the 3' terminus [19,20]. The FoMV genome structure resembles that of PVX, the type species of the genus *Potexvirus*, and the gene functions are presumed to be similar as well [21,22]. The genome of FoMV contains five open reading frames (ORFs), and two subgenomic promoters directing transcription of subgenomic RNAs (sgRNAs) 1 and 2 [21]. The genomic RNA allows the expression of ORF1 encoding the RNA-dependent RNA polymerase (RdRP) with methyltransferase, helicase, and polymerase motifs in PVX [23]. The first sgRNA contains ORF2, 3 and 4 coding for the triple gene block (TGB) proteins TGB1, TGB2 and TGB3, which are required for virus cell-to-cell movement [24]. The PVX ORF2 codes for a multifunctional protein that has RNA helicase activity, promotes translation of viral RNAs, increases plasmodesmatal size exclusion limits, and acts as a suppressor of RNA-mediated post-transcriptional gene silencing (PTGS) [24]. The PVX ORF5 encodes the coat protein, which is required for viral encapsidation, cell to cell movement, and long distance movement [25,26].

FoMV has a broad host range, infecting 56 species of the Poaceae and at least 35 dicot species [27]. The sequence of FoMV genomic RNA was first published in 1991 [21]. Infectious full-length clones were constructed based on the same FoMV isolate and some corrections to the published sequence were noted [28]. The significant difference between the gene organizations of FoMV and PVX is the presence of ORF 5A upstream of the CP gene in FoMV. ORF 5A initiates 143 nts upstream of the CP and extends the reading frame of CP gene. The 5A protein was produced in vivo, but it was not required for either replication or productive infection of plants [28]. Recently, the revised full-length sequence of foxtail mosaic virus clone was published in 2008, and reveals a triple gene block structure similar to *potato virus X*[22].

The potexvirus replicase is the only protein translated directly from the full-length genomic RNA, but other viral proteins are translated from 3' coterminal sgRNAs [29,30]. Two sgRNAs of approximately 2.1 and 0.9 kb in length have their 5' termini upstream of the TGB and CP genes, respectively [19], while a 1.4 kb bicistronic (readthrough) sgRNA provides for the translation of TGB2 and TGB3 ORFs [31]. The integrity of the subgenomic promoter in a vector is very important for the accumulation of sgRNA and target protein. However, the boundaries of sgRNA promoters have not been delineated for FoMV.

Vectors using FoMV have not been reported until this present study, but foundational work on potexvirus vectors has been completed with PVX. PVX was engineered to express reporter genes cloned just upstream of the CP gene and expressed from a duplicated copy of the CP subgenomic promoter [32]. The reporter gene was translated from a sgRNA separate from the other viral ORFs. Because PVX has a linear helical capsid, rather than an icosahedral capsid, a recombinant viral genome longer than wild type can still be encapsidated into infectious virus particles. Expression of longer ORFs with this construct led to earlier deletion of the ORF from the vector [33]. A PVX vector with a bicistronic sgRNA, carrying the reporter gene and the CP gene, has also been constructed. An IRES site allowed for translation of the distal gene on the sgRNA [34]. As mentioned previously, a deleted PVX vector has also been constructed [14]. A vector using another potexvirus, *Alternanthera mosaic virus*, has been recently developed [35]. In this vector system, the potexvirus was split into two components, the replicase portion and the TGB/CP portion, each in its own binary vector and *Agrobacterium *culture. Following co-agroinoculation, recombination in vivo regenerates the full length virus. In vivo expressed T7 RNA polymerase is used to transcribe the transgenic virus components after agroinoculation.

In this study, a vector with the properties of high protein expression and greatly lowered environmental risk was constructed. The TGB and CP genes of FoMV were removed and replaced with heterologous sequences while the subgenomic promoter of the first TGB gene (TGB1) was reserved to direct the transcription of the heterologous coding sequence. The FoMV expression vectors driven by the 35S promoter were delivered as a T-DNA to plant cells by agroinoculation. These severely crippled viral vectors would have difficulty surviving in the wild, since they form no virions, use agroinoculation for transmission, and are unable even to productively express marker genes unless a silencing suppressor is supplied. However, expression levels are among the highest of those reported from plant expression systems when silencing suppressor co-expression is provided.

## Results

### Construction and agroinoculation of full length JL22/pFoMV

**Figure 1:**
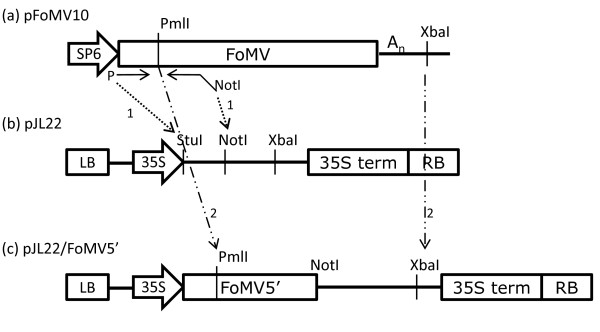
**Construction of pJL22/FoMV, the full length FoMV construct**. (a) Source in vitro transcription construct of native FoMV [28]. (b) Recipient binary vector, pJL22 [18]. A PCR fragment with a phosphorylated blunt upstream end and a NotI site at the downstream terminus was created by PCR from pFoMV10. This was ligated (1) into the StuI (blunt) and NotI sites of pJL22 to create pJL22/FoMV5'. (c) Next, the remainder of the FoMV sequence was added by creating a restriction fragment from pFoMV10 with PmlI/XbaI digestion. This was ligated (2) to the same sites in pJL22/FoMV5' to create pJL22/FoMV (Figure 3).

FoMV full length wild type viral cDNA from an in vitro transcription construct [26] was inserted into the binary vector, pJL22 [18] (Figure 1). To accomplish this, the PCR-amplified 5' end of the FoMV sequence was first transferred, followed by the insertion of a restriction fragment containing the rest of the FoMV sequence (Figure 1). The resulting construct (JL22/FoMV) comprised the 35S promoter, the full FoMV sequence, a poly(A) tract present in the original sequence [28], and the 35S terminator.

*A. tumefaciens *GV3101 was transformed with pJL22/FoMV and agroinoculated to *N. benthamiana*. At 1 and 2 weeks, there were no visible symptoms of viral infection on any plants. To detect the presence of FoMV infection, total RNA was extracted from leaves and screened by RT-PCR at 7 days post-inoculation (dpi). Systemic movement of FoMV was demonstrated in inoculated plants by the presence of RT-PCR product, but no bands were seen in uninoculated controls (data not shown). Thus, agroinfection with the JL22/FoMV construct resulted in replication and movement of this FoMV sequence but the infection proved very mild.

### Construction and agroinoculation of deletion vectors (FECT)

**Figure 2:**
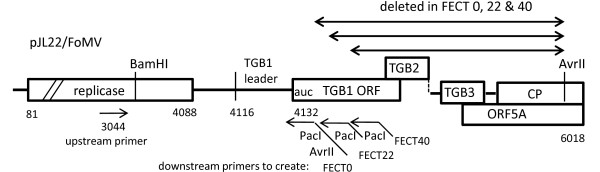
**Deletions of pJL22/FoMV that led to the construction of the FECT vector series**. Fragments containing various lengths of TGB1 subgenomic promoter were created by PCR between an upstream primer binding in the FoMV replicase region (nt. 3044) and a downstream primer which mutated the TGB1 AUG start codon to AUC and also added a PacI and AvrII site downstream of the AUC. This fragment was digested with BamHI and AvrII and inserted into pJL22/FoMV to take advantage of the native BamHI site in the replicase (3081) and the AvrII site 93 bases upstream of the CP ORF translational stop (stop at 6018) to create FECT0. FECT0 retains a subgenomic promoter consisting of the replicase 3' end and the TGB1 RNA leader but has no TGB1 ORF codons; it also retains 93 bases of the 3' end of the CP ORF. A PacI/AvrII cloning site is present after the TGB1 leader in FECT0 and subsequent FECT versions. FECT22 and FECT40 extend the potential TGB1 subgenomic promoter by an additional 22 and 40 bases, respectively, of TGB1 upstream ORF sequence. These were created from FECT0 with the upstream primer at 3044 and primers downstream of the AUC in FECT0. This fragment was digested with native BamHI and added PacI (contained in the sequence of the downstream primers) and inserted into these sites in FECT0.

**Figure 3:**
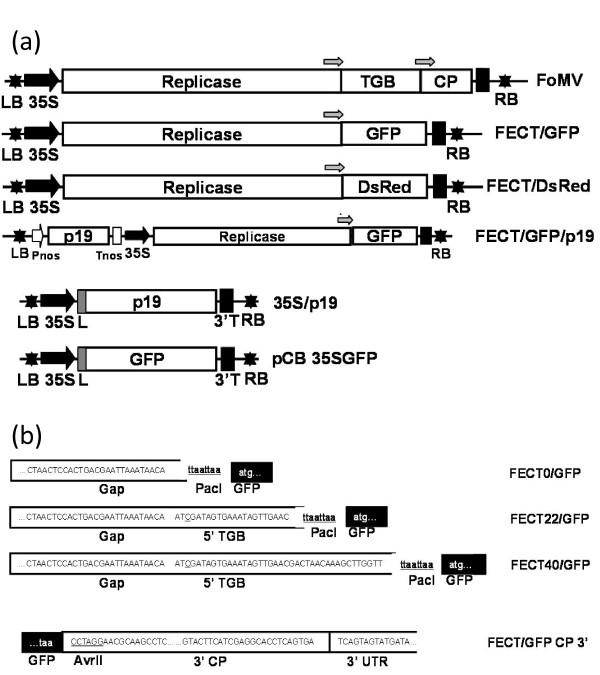
**Schematic diagram of (a) the T-DNA and (b) control regions of the FECT vector series**. (a) The native FoMV infectious sequence [28] was inserted into a JL22 binary vector as in Figure 1 and is represented here as "FoMV". The FECT series (Figure 2) constitutes the deleted FoMV vector. Various inserts were placed into the PacI/AvrII cloning site of FECT40 in this study. p19 silencing suppressor was added either in trans (via co-agroinoculation of a 35S/p19 construct) or in cis (in the same binary vector as FECT40, but with a separate promoter and terminator). Open boxes represent open reading frames; black stars: left border and right border of T-DNA; block arrows: CaMV duplicated 35S promoter; black boxes: CaMV 3' terminator sequence; gray arrows, subgenomic promoters; white arrow: nos promoter (Pnos); white box: nos terminator (Tnos); dark grey boxes: Tobacco etch virus 5' non-translated leader sequence (L); RB: T-DNA right border sequence; LB: T-DNA left border sequence; TGB: triple gene block; CP, coat protein. (b) Three different lengths of TGB1 subgenomic promoter were tested in their ability to drive GFP ORF expression. These were FECT0, FECT22 and FECT40 which included sequence extending 0, 22 and 40 bases, respectively from the first base of the TGB1 ORF. The start codon of TGB1 was mutated to ATC (underlined). Restriction sites PacI and AvrII were introduced at the flank of GFP ORF as cloning sites for other foreign inserts.

Primers were designed to delete the TGB and CP genes while retaining the subgenomic promoter of sgRNA1 and 3'-end of the CP gene to enable expression of foreign genes (Table 1; Figure 2). The extent of the sgRNA1 promoter region was unknown, so the entire sgRNA1 putative leader sequence and portions of the TGB1 ORF were included. Three upstream primers were used to include the first 0, 22 and 40 bases of ORF of TGB1 to create pFECT0, pFECT22 and pFECT40, respectively. The native TGB1 AUG was mutated to AUC and PacI and AvrII sites were included as cloning sites. The 3' terminal part of CP FoMV gene between AvrII and 3'- UTR was reserved because deletion of this region drastically reduced vector accumulation in a deleted PVX vector [14]. The final form of these constructs is diagramed in Figure 3.

**Table 1:** Primers used for vector construction.

Plasmid	Primer	Oligonucleotide sequence (5'-3')	Purpose
pFoMV/ JL22	FoMV 5' term UP (pFoMV nt. 1-21) FoMV756 NotI DOWN (pFoMV nt. 737-757)	P-GAAAACTCTTCCGAA ACCGAA TTTTTTGCGGCCGCTTAGC CAGTTTAGGTCCTTA	The 5' end of FoMV was amplified by PCR with primers FoMV5'termUP and FoMV756NotDown and cut with PmlI. The 3' end of FoMV was digested with PmlI and XbaI. Both 5' and 3' end fragments of FoMV were cloned into the JL22 backbone cut with StuI and XbaI.

pFECT0 pFECT22 pFECT40	FoMV Up (pFoMV nt 3044-3063) FoMV+0sgp Down (pFoMV nts. 4114-4131) FoMV+22sgp Down (pFoMV nts. 4124-4153) FoMV+40sgp Down (pFoMV nts. 4150-4169)	GTGGGCATGTGCAGATGA GG AACCTACCTAGGACTTTA ATTAATGTTATTTAATTCG TCAGTG GCTTTTAATTAAGTTCAA CTATTTCACTATCGATTGT TATT GTCTTTAATTAACCAAGC TTTGTTAGTCGTTC	To create ΔTGB/ΔCP mutants, PacI and AvrII cloning sites were introduced by PCR amplified with two primers (FoMVUp and FoMV+0sgp Down). PCR with mutated start codon of TGB was cut with BamHI and AvrII and cloned into pFoMV vector backbone to create pFECT0. Other two downstream primers (with PacI site) were used to save 22nts and 40nts 5' end of TGB DNA sequence. PCR fragments were cloned in pFECT0 vector backbone cut with BamHI and PacI to generate pFECT22 and pFECT40.

pFECT0/ GFP pFECT22/ GFP pFECT40/ GFP	PacGFPUp GFPAvrDown	TTGTCATTAATTAAGCTA GCAAAGGAGAAGAAC TTTACTCCTAGGTTATTTG TAGAGCTCATCCA	To clone the GFP ORF into the pFECT vector. Primer PacGFPUp adds a PacI site (underline) at the 5' end, and primer GFPAvrDown adds an AvrII site (underline) to the 3' end.

pFECT40/ GFP/ PnosTnos	ApaI Pnos UP PnosBsiWI- overlapDN TnosSpeI- overlapUP SbfI Tnos DN	ATATGAGGGCCCAACTGA AGGCGGGAAACGACAATC GACCACTTTATGGAGGTT CGTACGTCTAGGGGATCC GGTGCAG AACCTCCATAAAGTGGTC ACTAGTATCGTTCAAACA TTTGGC ATTATGCCTGCAGGAGCT GGCATGCAAGCTGTCGAGG	To add PnosTnos in pFECT40, and create BsiWI and SpeI in between Pnos and Tnos. Inner primers PnosBsi-overlapDN and TnosSpe-overlapUP have overlap sequence and BsiWI and SpeI sites. Two inner primers pair with outer primers ApaPnosUP (ApaI at 5' end) and SbfTnosDN (SbfI at 3' end) to generate two PCR products. The two products were fused using outer primers and cloned into pFECT/GFP.

pFECT40/ GFP/p19	BsiWI/p19 UP p19SpeI DOWN	TAATAACGTACGATGGAAC GAGCTATACAAG TTTTTTACTAGTTTACTCG CTTTCTTTTTCGAAGG	To clone the p19 ORF into pFECT40/GFP/PnosTnos vector. Primer Bsip19UP adds a BsiWI site (underline) at the 5' end and primer p19SpeDown adds a SpeI (underline) site at the 3' end of the ORF. The amplified DNA fragment was cloned into pFECT40/GFP/PnosTnos vector backbone cut with BsiWI and SpeI.

**Figure 4:**
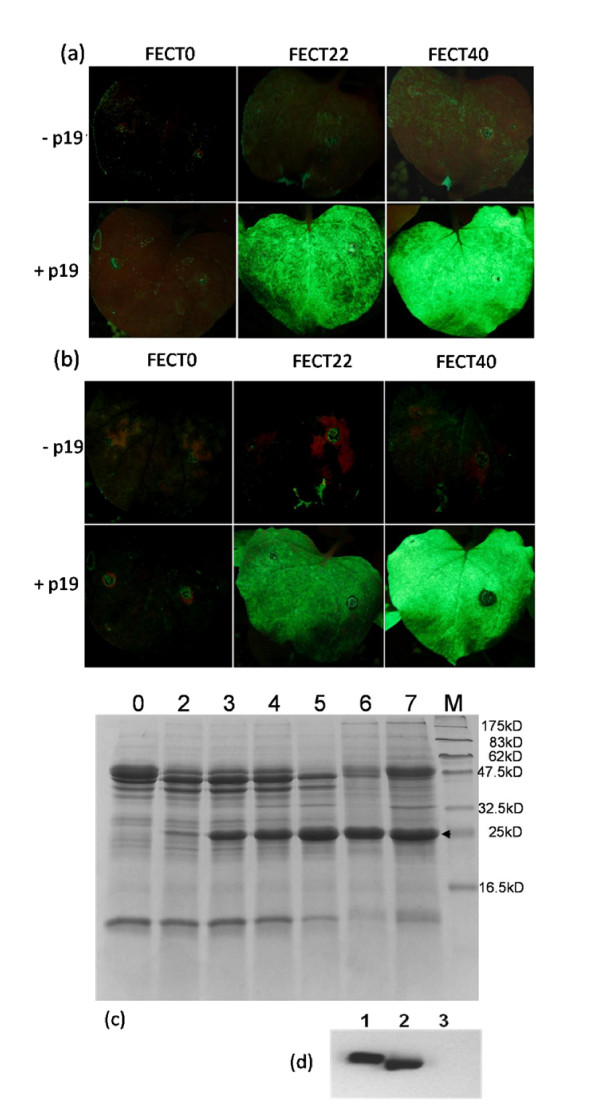
**GFP expression with differing subgenomic promoters and rescue by gene silencing suppressor coexpression**. (a) 4 dpi and (b) 8 dpi. In both panels, the top row show fluorescence from *N. benthamiana *agroinoculated with FECT/GFP but without p19 silencing suppressor and the bottom row shows leaves co-agroinoculation of FECT/GFP and 35S/p19. Only a small number of very faint fluorescent spots were found in FECT22 and FECT40 leaves at 4 dpi (a), and this fluorescence disappeared by 8 dpi (b). However, a vigorous fluorescence occurred with the addition of 35S/p19. FECT0 plants did not fluoresce even in the presence of 35S/p19. (c) FECT40/GFP was co-agroinoculated with 35S/p19 onto *N. benthamiana *and samples were taken each day of the time course and protein extracted by grinding and centrifugation. Lanes: M, protein marker; 0, protein extract from uninoculated leaf; 2 to 7, extracts from FECT agroinfiltrated leaves, 2 to 7 dpi, respectively. (d) Western blot of GFP expression at 7 dpi. Lanes: 1, 0.2 μg GFP standard (*E. coli *generated); 2, FECT/GFP + 35S/p19 in *N. benthamiana*; 3, 35S/p19 alone in *N. benthamiana*.

To test viral replication and foreign gene expression, GFP was inserted into each of the FECT vectors (Figure 3). *N. benthamiana *was agroinoculated with GV3101 *Agrobacterium *cultures carrying FECT. At 2-4 days after agroinoculation, GFP-expressing cells could be seen faintly using a hand-held UV lamp and fluorescence microscopy. At this time, there were many faint green spots showing on leaves inoculated with FECT40/GFP and FECT20/GFP (but fainter in the latter), but no green fluorescence could be detected on leaves inoculated with FECT0/GFP (Figure 4a). Furthermore, the fluorescence was transient and, by eight days post-inoculation, the GFP spots on all plants had disappeared (Figure 4b). Apparently, the transcription of agroinfiltrated T-DNA induced posttranscriptional gene silencing (PTGS), which led to the inhibition of viral vector infection and the reduction of viral productivity [36].

### Rescue with silencing suppressors

It has recently been demonstrated that co-inoculation of RNA silencing suppressor proteins enhances the expression of heterologous proteins from viral vectors [14,18]. To test this effect, *N. benthamiana *plants were agroinfiltrated with a 1:1 mixture of 35S/p19 or 35S/HcPro and FECT/GFP cultures. The accumulation of GFP was followed and imaged with a hand-held UV light and fluorescence microscopy for 3-7 days post-inoculation.

When plants were co-infiltrated with the suppressor, the level of fluorescence was surprising (Figure 4a and 4b). The fluorescence of the inoculated zones of FECT40/GFP plants was very clearly seen under the UV lamp even with the room lights turned on (data not shown). FECT22/GFP plants, though quite fluorescent, were clearly less so than FECT40/GFP inoculated plants. Furthermore, no fluorescence was seen with FECT0/GFP with or without suppressor co-infiltration (Figure 4a and 4b). Ds-Red was also expressed with FECT40 with similar results (data not shown).

### Quantification and comparison to other vectors

The unusually high expression level led us to quantify the percent of total soluble plant protein that the GFP represented in the inoculated zone. Over the course of two weeks, fluorescence had appeared to increase through the first week and then stabilize so a time course assay covering the first week was initiated. *A. tumefaciens*/FECT40/GFP + *A. tumefaciens*/35S/p19 co-infiltrated leaves from 2 to 7 dpi were homogenized and the relative amounts of GFP in extracts of total soluble protein were measured with SDS-PAGE electrophoresis and Coomassie blue protein staining (Figure 4c). GFP expression was detected from the second day after inoculation (Figure 4c). The expression level of fluorescent protein increased gradually, and stabilized at 5-7 dpi (Figure 4c). GFP accumulated to 30% to 40% of the total soluble protein extracted, as measured by densitometry.

**Figure 5:**
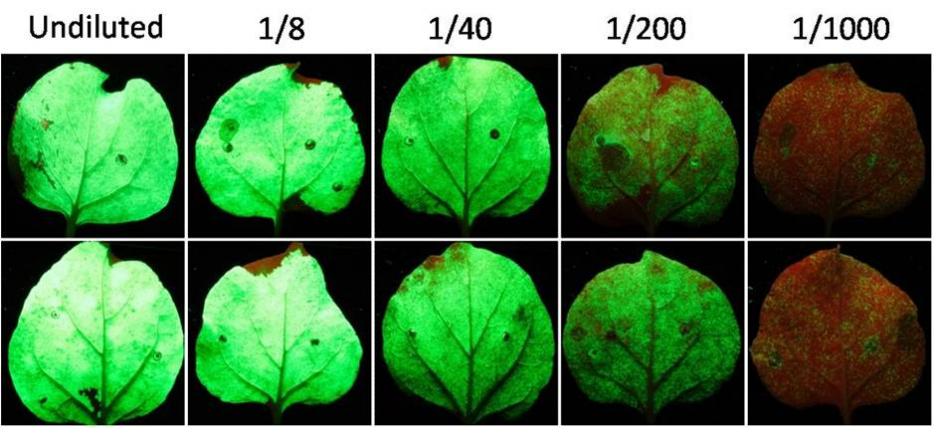
**Dilution trial of agroinoculant with FECT and p19 on *N. benthamiana *plants**. Top row: FECT40/GFP vector. Bottom row: JL24/GFP vector. *A.t*. cell suspensions were diluted, as noted in the figure, from an initial OD600 of 1.0. Pictures were taken under UV illumination at 4 dpi with photos taken at same exposure.

**Figure 6:**
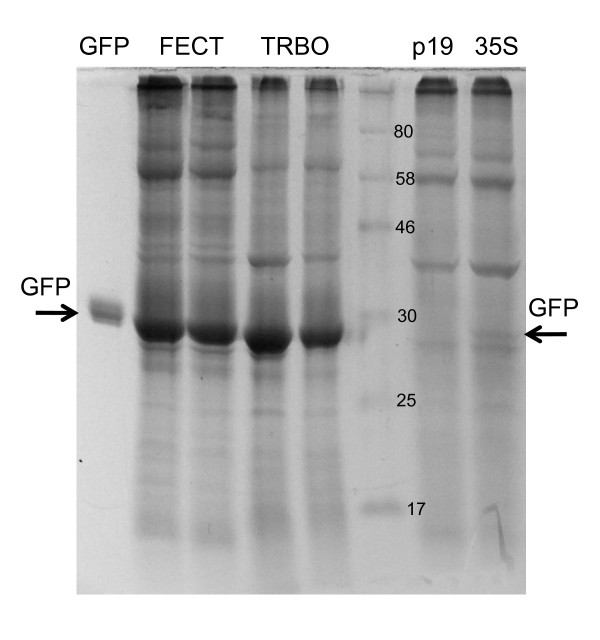
**Comparison of FECT40 GFP expression with TMV and 35S vectors in *N. benthamiana*, 7 dpi**. Lane 1, 1 μg GFP standard, Lanes 2 & 3, FECT40/GFP + 35S/p19, Lanes 3 & 4, TRBO/GFP + 35S/p19, Lane 5, NEB prestained broad-range protein markers, Lane 6 35S/p19 alone, Lane 7, 35S/GFP (pGDG vector) + 35S/p19. Each sample lane represents approximately 5.5 mg fw of plant tissue.

This expression level was further quantified and compared to TMV vectors, which are the most commonly used plant viral expression vectors. In a dilution comparison with the full length TMV vector, JL24 [18], the expression of FECT40/GFP (top row) matched that of JL24 (bottom row) when both were co-agroinoculated with p19 (Figure 5). Thus, even with the disadvantage of no systemic movement, FECT vector expression was equivalent to that of an excellent systemic TMV vector. FECT40/GFP expression was also equal to the TRBO-G vector [13], which is among the most strongly expressing of the deleted TMV vectors. In SDS-PAGE/Coomassie blue densitometry analysis (Figure 6), FECT40 yielded GFP at 40% of total soluble protein (as for Figure 4c). The mg of GFP produced per gram fresh weight (gfw) of tissue was determined for FECT40/GFP by comparison to a GFP standard and was 1.6-1.7 mg/gfw, similar to that of TRBO-G with 1.3-1.7 mg/gfw (Figure 6). In comparison, the single-enhancer 35S promoter driving GFP expression in the standard binary vector pGDG [37], induced by p19 expression, yielded GFP at only 0.03 mg/gfw, or more than 50-fold less than FECT/GFP expression (Figure 6).

In support of these values were calculations made by western blot analysis. The GFP band of Figure 4d represented a 10 μl load from a 100× dilution of 300 μl of extract from 150 mg of fresh leaf material. The protein content of the virus-expressed GFP band was estimated as 0.2 μg by western blot, because it has the same density as the 0.2 μg GFP standard generated by a bacterial expression system. From these data we again determined a yield of 1.6 mg/gfw of GFP for FECT40/GFP. To further support this, GFP from FECT40/GFP infected tissue from three replicates from another experiment was quantified by spectrophotometry in comparison with bacterially-produced GFP standard. By this method, 1.58 ± 0.13 mg/gfw was determined for the GFP yield (data not shown).

### Expression in monocots and legumes via agroinoculation

**Figure 7:**
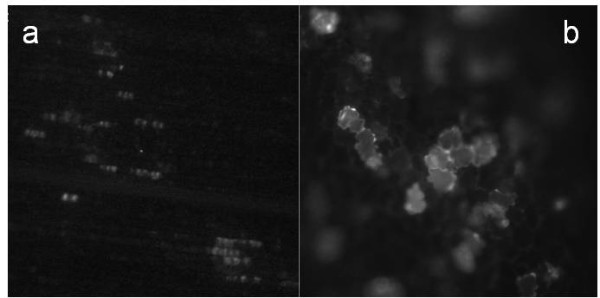
**Fluorescence microscopy of monocots and legumes agroinoculated with FECT40/GFP/p19**. (a) Maize leaf infection. For all grasses tested, occasional areas of scattered fluorescent cells were seen more commonly in FECT40/GFP/p19 agroinoculated leaves than in p19 agroinoculated or noninoculated leaves, but such cells were rare nonetheless. (b) *Medicago trunculata *leaf infection. Scattered fluorescent cells were common and FECT40/GFP/p19-infected tissue was easily distinguishable from p19-inoculated control leaves under fluorescent microscopy for both *M. trunculata *and lentils.

Since the natural host range of FoMV includes many grass and legume species, it was appropriate to test GFP expression of FECT40/GFP plus 35S/p19 via agroinoculation against a panel of grass and legume species via agroinoculation. Switchgrass, foxtail millet, barley, wheat, oat and maize were co-agroinoculated with the mixture of two *Agrobacterium *cultures containing FECT40/GFP and p19, respectively. Patches of widely spaced fluorescing cells were observed by UV microscopy in all grass species (Figure 7a) beyond the few number of autofluorescent cells in uninoculated controls. However, these isolated fluorescent cells were still quite uncommon in inoculated grass leaves. A C58 strain of *A. tumefaciens *carrying FECT40/GFP was also tried, but did not provide any improvement in expression rates in grass species. In the legume, *M. trunculata*, however, relatively larger numbers of isolated fluorescing cells were commonly encountered in inoculated tissue (Figure 7b), while few or no autofluorescent cells were seen in uninoculated controls. Somewhat fewer fluorescent cells were found in lentils, while no strong evidence for infected cells was found in bean or cowpea by fluorescence microscopy.

**Figure 8:**
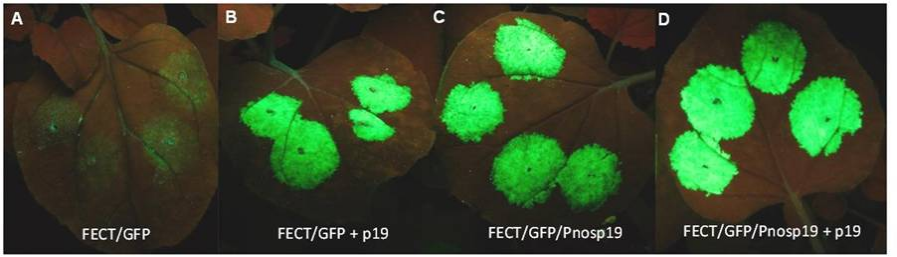
**Effect of p19 on GFP expression of FECT/GFP in trans and cis formats in *N. benthamiana***. GFP fluorescence at 2 dpi as seen under UV light for the standard 35S-driven FECT40/GFP co-agroinoculated with 35S/GFP (trans) or for a binary vector containing both 35S/FECT40/GFP and Pnos-driven p19 driven (cis). The cis format was constructed in an attempt to better visualize FECT40/GFP expression in plants with poor rates of agroinoculation (such as monocots), to eliminate the need for double infection by both p19 and FECT40/GFP.

We have demonstrated in *N. benthamiana *that expression of FECT/GFP requires p19 co-expression and we expect that most cells are doubly infected with agrobacteria containing FECT/GFP or 35S/p19. However, in the agroinoculation of grasses, only a small fraction of cells, at best, are expected to be even singly infected by *Agrobacterium *[38]. Even fewer cells would be expected to be doubly infected with two different agrobacteria containing FECT/GFP and p19, respectively, and this might explain the difficulty in visualizing fluorescent cells in grasses. For this reason, we added to the binary vector carrying 35S/FECT/GFP another component carrying the *nos *promoter (Pnos) driving the expression of p19 and terminated by the *nos *terminator (Tnos). This cis-acting construct tested very strongly in *N. benthamiana *at 2 dpi (Figure 8), demonstrating an independence from p19 co-agroinoculation for strong fluorescence in the cis construct. By 6 dpi, the fluorescence of the trans configuration (separate binary vectors carrying FECT/GFP and 35S/p19) had increased so as to be somewhat superior to the cis configuration. In spite of these *N. benthamiana *results, when the cis-configured FECT/GFP/p19 was co-agroinoculated onto various grass species, no increase over the trans configuration in fluorescent cell numbers or brightness was seen either by eye or by fluorescence microscopy.

## Discussion

We have demonstrated very high protein expression rates in *N. benthamiana *for the deleted viral vector, FECT, in co-agroinoculation with 35S/p19. At 40% TSP and 1.6 mg/gfw for the expression of GFP, FECT expresses well beyond expression rates published for traditional plant virus vector systems [32,39]. The GFP expression of the best nonviral 35S system is 270-340 μg GFP/gfw aided by p19 and 5-12 μg/gfw without p19 [36]. The most recently developed TMV vectors, TRBO [13] and the magnifection system [10] both report yields of up to 5 mg/gfw. In side-by-side comparison, with p19 co-inoculation for both, however, FECT expressed GFP at levels equal to those of TRBO. Thus, we have developed a simple and highly efficient protein synthesis vector system for plant-based expression.

The FECT viral vector system has many features that enhance its environmental safety when used via agroinoculation or potentially as a transgene. First, its genome is reduced by almost one-third, making it severely crippled. Second, it does not replicate efficiently unless the plant immune system is suppressed. Thus, if it were to somehow infect another plant (e.g., via its *Agrobacterium *carrier), it would replicate poorly. Third, it lacks a coat protein and cannot form a virion, making it improbable for the virus to survive outside of a host. Finally, it is derived from a virus that in most hosts causes mild infections [27]. In fact, we observed no symptoms in *N. benthamiana *with the full length virus.

We observed a novel control mechanism operational with the FECT construct, which could be very useful in further applications of FECT. In the absence of a silencing suppressor, almost no expression of GFP occurred, and what expression did occur was transient, disappearing by the seventh day after inoculation. This contrasts with the extreme expression in the presence of the silencing suppressor. This was not the case with the PVX vector of Komorova et al. [14]. Though PVX and FoMV are both potexviruses, the Komorova deletion vector performed quite well, better than the full length standard PVX vector, even in the absence of suppressor. Furthermore, this paper states that no extra bases of ORF were used to extend the subgenomic promoter, but we found no expression when we emulated this with FoMV. Only when extra bases of the TGB1 ORF were included was expression seen, and for significant expression, suppressor co-expression was needed. Thus, the vectors differed in design as well as performance.

Tyulkina et al. [40] examined the effect of removing the coat protein of TMV (tobamovirus family) and portions of the movement protein. They found that complete removal of the movement protein produced only small infection points, which could be rescued by the co-agroinoculation of p19 or of various sized portions of the TMV movement protein. Thus, a similar effect was found in the tobamoviruses as we found with the FoMV potexvirus. In contrast, the work of Komorova et al. [14] appeared to clearly demonstrate that, for potexviruses, the removal of coat protein and triple gene block genes produced a vector which was quite functional in the absence of suppressor. Our results with the FoMV potexvirus were thus unexpected.

There may be functional differences in the replication mechanisms between FoMV and PVX which are unexplored at present. For example, major differences in silencing suppressor activity of TGB1 have been found between different potexviruses [41] and even between variant sequences of a single potexvirus, *Alternanthera mosaic virus *[42]. Though TGB1 was deleted in both the FoMV and PVX vectors, it may be that other features in these viruses, affecting their ability to replicate when the TGB1 silencing suppression is eliminated, may also exhibit taxonomic diversity. It may be further speculated that host proteins that aid potexvirus replication, such as NbPC1P1 [43], might be more critical for some potexviruses than for others. The omission of, for example, the CP gene, which NbPC1P1 binds to, might be more detrimental for FoMV than for PVX.

The very low rate of infectivity of FECT in grasses is not unexpected. It is for this reason that full virus vectors, capable of obviating low agroinfection rates via cell-to-cell and long-distance movement, are used for agroinoculation studies [44,45]. There is only one paper to our knowledge that demonstrates successful visualization of single cell agroinfections of grasses [38]. In this work, only scattered cells transformed with a 35S/GUS construct were visualized. We were unable to utilize the GUS marker gene in this study because of GUS production in the agrobacterium culture itself from the FECT/GUS construct (data not shown). However, the creation of FECT/GFP/Pnosp19 opens the possibility of the use of FECT as a viral transgene in grasses, since both p19 and FECT would be expressed in each cell.

## Conclusions

There are several potential applications for the use of FECT vectors. This system has the capacity for high level expression of a variety of proteins, including GFP (0.7 kb), the larger GUS (1.8 kb) and the multimeric Ds-Red (0.7 kb) proteins. The inability of FECT to replicate significantly in the absence of silencing suppression or to survive or infect in unencapsidated form greatly reduces environmental risk. This system, then, would be expected to be amenable to the production of pharmaceutical or industrial proteins via agroinoculation in the greenhouse. The tight on/off control of this system also makes it suitable for an inducible transgenic system for field use and for the expression of proteins toxic to the plant host. Finally, the use of FECT as an agroinoculation vector for screening genes and gene variants for plant expression before the construction of stably transgenic plants is facile due to high expression and lack of cross contamination of greenhouses and growth chambers.

## Methods

### Plants

All plants were grown in a dedicated plant growth room with temperatures ranging from 22-24°C and with automated watering. All seeds were sprouted under plant spectrum fluorescent bulbs. *N. benthamiana *seedlings were then transplanted and grown for two weeks under 400 W metal halide lamps to 10-15 cm before agroinoculation. *Panicum virgatum *cv. Blackwell (switchgrass), *Setaria viridis *(foxtail grass), *Hordeum vulgare *(barley), *Triticum aestivum *(wheat), *Avena sativa *(oat) and *Zea mays *(corn), *Medicago trunculata *(barrel medic), *Lens culinaris *(lentil), and *Cicer arietinum *(chickpea) plants were germinated from seed and grown with 24h/day illumination with plant spectrum fluorescent bulbs. Grasses and legumes 2-3 weeks from seed, with fully expanded leaves, were used for agroinoculation.

### Vector construction

All FoMV viral cDNA constructs used in this study are derivatives of a wild-type FoMV cDNA clone that was a gift from Nancy Robertson of the USDA [28] and were constructed with standard recombinant DNA techniques. The binary vector, pJL22, provided by John Lindbo [18], has the mini binary plasmid, pCB301 [46], as a backbone. JL22 contains multiple cloning sites flanked by a 35S promoter and 35S polyA signal/transcription terminator (Figure 1). The source FoMV sequence had an additional 70 adenosine residues inserted after the viral 3' terminus, followed by a XbaI site. To create JL22/FoMV, the 5' end of FoMV was amplified by PCR with primers FoMV5'termUP and FoMV756NotDown and cut with NotI. The 5' end of FoMV was cloned into JL22 digested with StuI and NotI to create JL22/FoMV5'. The 3' end fragment of FoMV was prepared by restriction digest with PmlI and XbaI and then inserted into JL22/FoMV5' also digested with PmlI and XbaI (Figure 1). pJL22 contains a CaMV 35S polyA signal to generate a poly(A) end, following the poly(A) end already included in the Robertson sequence. This full viral cassette including promoter and terminator is flanked with Left Border and Right Border of the T-DNA ("FoMV", Figure 3).

The FECT vector series was created from JL22/FoMV using PCR to delete the TGB and CP genes. To ensure that the full sgRNA1 promoter was retained, primers were created which included the first 0, 22 and 40 bases of ORF of TGB1 to create pFECT0, pFECT22 and pFECT40, respectively. PacI and AvrII sites were placed directly after the retained sgRNA1 promoter of TGB1 for insertion of foreign ORFs. The downstream primer used to make FECT0, namely, FoMV+0sgpDown, mutated the native TGB1 AUG start codon to AUC and added both AvrII and PacI sites at 3' end of subgenomic promoter TGB1. Upstream primer FoMVUp is upstream of a unique native BamHI site in FoMV (Figure 2). With these two primers, a PCR fragment was created and was digested with BamHI and AvrII and cloned back into JL22/FoMV cut with BamHI and AvrII to create pFECT0. Since a native AvrII site was present at nt. 5925, 93 bases upstream from the end of the CP ORF (5371-6018) (Figure 2), inserting this PCR fragment into JL22/FoMV at the BamHI and AvrII sites enabled all three TGB ORFs and most of the CP ORF to be deleted in one step to create FECT0. The 3' terminal part of CP FoMV gene between AvrII and 3'- UTR was reserved for efficient expression. To create vectors with longer subgenomic promoters, two primers, FoMV+22sgp and FoMV+40sgp, were paired with FoMVUP to generate two PCR fragments including 22 and 40 bases of TGB1 ORF, respectively. These PCR fragments were digested and inserted back into pFECT0 cut with BamHI and PacI to create pFECT22 and pFECT40. To insert GFP, the GFP ORF was amplified using primers containing either PacI or AvrII sites (Table 1) and the PCR product was digested and inserted into the PacI/AvrII cloning site in FECT (Figure 3).

The high fidelity polymerase, Phusion (New England Biolabs (NEB), Beverly, MA), was used according to company protocols in all constructions. Recombinant clones were introduced into *E. coli *10-beta electrocompetent cells (NEB, Beverly, MA) by electroporation at 1.44kV and 129 Ω for 5 ms using a BTX 600 Electro Cell Manipulator (BTX Inc., San Diego, CA, USA) and colonies were screened by PCR using NEB Taq polymerase or by restriction digests of plasmid minipreps prepared by Wizard Plus Miniprep Kit (Promega, Madison, WI). Sequence verification was performed using a CEQ capillary sequencer (Beckman Coulter, Fullerton, CA).

### Agroinoculation

Agroinfiltration was performed as described [47] with modifications. *Agrobacterium tumefaciens *stain GV3101 was used for the agroinoculation of *N. benthamiana *and cereals. *A. tumefaciens *was transformed with plasmid constructs using the same conditions as for *E. coli *above. *Agrobacterium *transformants were selected at room temperature on Luria-Bertani plates containing 10 μg/ml rifampicin, 25 μg/ml gentamycin and 50 μg/ml kanamycin. A colony of *A. tumefaciens *was inoculated to 5 ml of L-MESA medium (LB media supplemented with 10 mM MES, 20 μM acetosyringone (Phytotechnology Labs, Shawnee Mission, KS)) and the same antibiotics, and grown overnight at room temperature. The cells of the overnight culture were harvested by centrifugation and resuspended in induction media (10 mM MES, 10 mM MgCl_2_, 100 μM acetosyringone) to a final OD_600 _of 1.0 and incubated for 2 h to overnight at room temperature. The cultures of *A. tumefaciens *were infiltrated into the underside of attached leaves with a 3 ml syringe without needle. For co-agroinoculation of two or more bacterium cultures, cultures of *A. tumefaciens *were mixed in equal amounts and infiltrated together. The gene expression or virus activity was tested at 6-8 days post-infiltration and one of three plant replicates were analyzed per experiment.

### RT-PCR

To detect FoMV (without GFP or DsRed) in the plant, total RNA was extracted after 7 dpi using Tri-Reagent (Sigma, St. Louis, MO) according to the manufacturer's protocol. RT-PCR reactions were performed using the RT-PCR kit (NEB, Beverly, MA) as described by the supplier. To detect the presence of virus sequence, FoMV specific primers were used to amplify the partial viral genome.

### GFP and DsRed photography

Plants were examined under long-wave UV light (UVL-56, UVProducts, Upland, CA). For macrophotography, a Canon Digital EOS Rebel XT camera (Canon Inc., Japan) equipped with a Hoya yellow (K2) filter (Hoya Corp., Japan) was used. For microscopic analysis, samples from infiltrated tissues were mounted with water on a glass slide. Images were obtained with a Nikon TE2000-U inverted microscope, captured using a CoolSnap *cf *camera (Roper Scientific, Tucson, AZ) and analyzed with Metavue imaging software (version 5, Molecular Devices Co, Downingtown, PA).

### GFP Quantification Assay

GFP fluorescence was analyzed and GFP protein was quantified using a standard curve determined from a purchased GFP standard (Vector Laboratories, Inc, Burlingame, CA), since the amount of GFP protein is directly proportional to the fluorescence intensity [18,48]. Total soluble protein extracts were serially diluted in 50 mM carbonate/bicarbonate buffer, pH 9.6 and loaded on the 96-well Costar black plate with clear bottom (Costar, Cambridge, MA). Fluorescent activities were assayed with a Fluoroskan Ascent FL (Thermo Fisher Scientific Inc., Waltham, MA) using a 485 nm excitation and 538 nm emission filter set.

### Protein extraction, SDS-PAGE

Proteins were extracted by grinding agroinoculated leaves to a fine powder in liquid nitrogen and mixing 1:2 (w/v) with reducing protein extraction buffer (50 mM tris, pH7.5, 150 mM NaCl, 0.1% Tween 20, and 0.1% β-mercaptoethanol) or nonreducing protein extraction buffer without β-mercaptoethanol. The insoluble material was removed by centrifugation for 10 min at 16,000 × g in a benchtop centrifuge. The supernatant was collected and stored at 4°C. Clarified extract of protein samples were mixed with denaturing 3 × SDS-PAGE sample buffer (NEB, Beverly, MA) and analyzed by PAGE consisting of a 5% stacking gel and a 7.5% or 15% separation gel. Proteins in the gels were identified with Coomassie brilliant blue R-250 (Sigma, St Louis, MO).

### Western Blot

Recombinant GFP detection was tested by western blot using total soluble protein (1 μg protein from FECT:GFP + p19 infections or 1 μg protein from p19 alone infections) and 2 μg bacterially expressed recombinant GFP (Victor Laboratories, CA) run on a precast linear gradient polyacrylamide gel (4-15%) (Bio-Rad, Hercules, CA). The gel was electroblotted onto a PVDF membrane (Amersham Biosciences, Piscataway, NJ) by a semi-dry transfer cell (Bio-Rad, Hercules, CA). The blot was blocked with TBST buffer (100 mM tris-HCl, 0.9% NaCl, 0.1% Tween 20, pH 7.5) containing 5% fat free milk at room temperature for 1 h. The membrane was incubated with an anti-GFP antibody (Invitrogen, Carlsbad, CA) (1 μg/ml) at room temperature for one hour. Bound rabbit IgG was detected by horseradish peroxidase-conjugated goat anti-rabbit IgG (Santa Cruz Biotechnology, CA). Visualization was performed via ECL detection reagents (Pierce). The gel image was taken by FluorChem SP imaging system (Alpha Innotech, San Leandro, CA) and the relative protein concentrations were analyzed by AlphaeaseFC software version 4.1.0 (Alpha Innotech).

## Abbreviations

dpi: days post-inoculation; ELISA: enzyme linked immunosorbent assay; FECT vector: FoMV eliminated CP and TGB; FoMV: *Foxtail mosaic virus*; CP: coat protein; GFP: green fluorescent protein; gfw: gram fresh weight; GUS: glucuronidase; MCS: multiple cloning site; ORF: open reading frame; Pnos: promoter of nopaline synthestase gene; PVX: *Potato virus X*; RT-PCR: reverse transcription polymerase chain reaction; SDS-PAGE: sodium dodecyl sulfate polyacrylamide electrophoresis; sgRNA: subgenomic RNA; T-DNA: transfer DNA; TGB: triple gene block; Ti: tumor-inducing; TMV: *Tobacco mosaic virus*; Tnos: terminator of nopaline synthestase gene; UTR: untranslated region.

## Authors' contributions

ZL carried out the construction of the vectors, the inoculation of plants, protein quantification, and drafted the manuscript. CMK conceived of the study, carried out the vector comparison, cis-configuration and microscopy studies, performed the photography and edited the manuscript. Both authors read and approved the final manuscript.
